# Chiral 3D Perovskite Single Crystals Realized by Lattice Expansion

**DOI:** 10.1002/advs.202506902

**Published:** 2025-07-01

**Authors:** Lin Wang, Wei Hao, Shen Chen, Jie Ren, Hanying Li

**Affiliations:** ^1^ MOE Key Laboratory of Macromolecular Synthesis and Functionalization, International Research Center for X Polymers, ZJU‐YST Joint Research Center for Fundamental Science Department of Polymer Science and Engineering Zhejiang University Hangzhou 310027 China

**Keywords:** chiral 3D perovskite, circularly polarized light, photodetector, second harmonic generation, single crystals

## Abstract

Chiral 3D perovskites possess remarkable chiroptoelectronic properties, which exhibit potential for overcoming the intrinsic limitations of carrier/exciton dynamics in low‐dimensional chiral perovskites. However, chiral 3D perovskites face significant synthetic challenges compared to their low‐dimensional counterparts, owing to the large steric hindrance of available chiral organic ammonium cations. Here, a novel chiral 3D perovskite single crystal, [(*R*/*S*)‐3APr]_2_Pb_4_I_12_·2H_2_O [(*R*/*S*)‐3APr = (*R*/*S*)‐3‐aminopyrrolidine] is demonstrated. The chiral ligands along with water molecules are incorporated inside 3D inorganic frameworks with expanded crystal lattices. An electrode‐transfer device fabrication strategy is developed to construct the chiral 3D perovskite‐based circularly polarized light (CPL) photodetector. The resulting photodetector exhibits excellent performance, with a high anisotropy factor for photocurrent *(g_I_*_ph_) of 0.4. Furthermore, the non‐centrosymmetry enables chiral 3D perovskites to have efficient second harmonic generation (SHG) properties and a large circular polarization sensitivity of SHG, with an optical anisotropy factor of 0.83, extending the CPL detection range to near infrared region.

## Graphical abstract

A novel chiral 3D perovskite [(*R*/*S*)‐3APr]_2_Pb_4_I_12_·2H_2_O single crystal is reported. This structure can accommodate large chiral diammonium cations while maintaining 3D skeletons by modulating the connectivity of [M_2_X_10_] dimers. The chiral 3D perovskite photodetectors exhibit notable circularly polarized light (CPL) detection performance. By leveraging the second‐harmonic generation effect, the CPL detection range is extended to near infrared region.

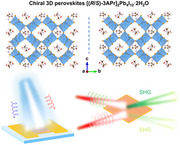

## 1 Introduction

Organic‐inorganic hybrid perovskites (OIHPs) have emerged as promising functional materials for optoelectronics due to their flexible crystal structures, long carrier‐diffusions lengths and tunable optical properties. The most extensively studied three‐dimensional (3D) perovskites adopt the general chemical formula of ABX_3_ (A = Cs^+^, CH_3_NH_3_^+^ (MA^+^) or HC(NH_2_)^2+^ (FA^+^); B = Pb^2+^ Ge^2+^ or Sn^2+^; X = I^−^, Br^−^, or Cl^−^), where the 3D crystal structure are formed by corner‐shared [BX_6_] octahedrons with A cations occupied the framework cavities.^[^ ^1^, ^2^ ^]^ By incorporating relatively large chiral cation into the A‐site, serving as spacers ligands, the 3D structures can be broken down and various lower‐dimensional chiral perovskites are formed,^[^ ^3^, ^4^, ^5^ ^]^ yielding chirality and unique chiroptical properties including circular dichroism (CD),^[^ ^6^, ^7^, ^8^, ^9^ ^]^ circularly polarized luminescence^[^ ^10^, ^11^ ^]^ and nonlinear optical (NLO) properties such as second harmonic generation (SHG).^[^ ^12^, ^13^ ^]^ These attributes have significantly promoted the expansion of the applications of OIHPs, rendering chiral perovskites as promising candidates for the chiroptoelectronic,^[^ ^14^, ^15^ ^]^ chiro‐spintronic,^[^ ^16^, ^17^, ^18^ ^]^ ferroelectric^[^ ^4^, ^19^ ^]^ and nonlinear optic fields.^[^ ^20^, ^21^ ^]^

The performance of perovskite devices strongly relies on the perovskite's crystalline structure and orientation. Typical low‐dimensional chiral perovskites, such as (*R*/*S*‐*α*‐PEA)PbI_3_,^[^ ^9^ ^]^ (*R*/*S*‐*α*‐PEA)_2_PbI_4_^[^ ^22^ ^]^ and (*R*/*S*‐3AMP)PbBr_4_,^[^ ^4^ ^]^ show intrinsic limitations such as high exciton binding energy and poor charge carrier transport, especially in the out‐of‐plane direction. In contrast, 3D perovskites have crystal structures interconnected by halides in the direction of three spatial axes, exhibiting distinct advantages, with long exciton lifetime^[^ ^23^, ^24^ ^]^ and high carrier mobility without dimensional restrictions.^[^ ^25^ ^]^ The synthesis of chiral 3D perovskites offers a promising strategy to realize advanced performance in chiral optoelectronic devices.^[^ ^26^, ^27^ ^]^ However, the controllable synthesis of chiral 3D perovskites remains a challenge, as suitable chiral cations need to be selected with desirable structure tolerance factor.^[^ ^28^, ^29^ ^]^ Although thermodynamically and kinetically stabilized chiral 3D perovskites have been predicted via theoretical calculations,^[^ ^30^ ^]^ experimentally explorations have rarely to be reported. Therefore, it is crucial for the development of construction strategies of novel chiral 3D perovskites, which is strongly demanded for high‐performance circularly polarized light (CPL) photodetectors.

Besides, numerous research efforts have been made in achieving direct CPL detection utilizing chiral OIHPs.^[^ ^31^, ^32^, ^33^ ^]^ However, CPL detection is mainly accessible in the ultraviolet (UV) or visible (vis) spectral regions and demonstrate a pronounced dependence on the intrinsic semiconducting properties of the materials.^[^ ^34^, ^35^ ^]^ The pivotal challenge in CPL detection in chiral perovskite‐based devices lies in the near‐infrared (NIR) spectral region,^[^ ^36^ ^]^ which holds significant potential for applications in night vision systems^[^ ^37^ ^]^ and autonomous vehicle technologies.^[^ ^38^ ^]^ Accordingly, it is imperative to devise novel methodologies to address this dilemma, enabling direct NIR CPL distinguishment that independent of the inherent absorption limitations.

Herein, we reported a novel chiral 3D perovskite single crystal [(*R*/*S*)‐3APr]_2_Pb_4_I_12_·2H_2_O [(*R*/*S*)‐3APr = (*R*/*S*)‐3‐Aminopyrrolidine]. Compared with the typical 3D perovskite structure, the inorganic frameworks are distorted and expanded, which accommodate large divalent 3Apr^2+^ cations and solvent molecules H_2_O. To eliminate the inevitable influence of vacuum thermal evaporation on the material and evaluate the electrical properties accurately, an electrode‐transfer methodology was developed for the device assembly of [(*R*)‐3APr]_2_Pb_4_I_12_·2H_2_O based photodetectors. CPL photodetector based on the chiral 3D perovskite single crystal exhibits superior CPL discrimination ability and excellent device performance, with a responsivity of 7.2 A W^−1^, a detectivity exceeding 9.4 × 10^12^ Jones and an anisotropy factor of 0.4. Circularly polarized‐dependent SHG intensity for [(*R*)‐3APr]_2_Pb_4_I_12_·2H_2_O was also investigated. By employing the SHG circular dichroism (SHG‐CD) technology, the optical activity range of [(*R*)‐3APr]_2_Pb_4_I_12_·2H_2_O for CPL sensing expands to the NIR region. [(*R*)‐3APr]_2_Pb_4_I_12_·2H_2_O exhibited a distinct SHG response under left‐ and right‐handed of the circularly polarized (LCP and RCP) laser pump ranging from 900 to 2000 nm. An impressive high anisotropy factor for circular polarization sensitivity (*g*_SHG‐CD_) of 0.83 was achieved for [(*R*)‐3APr]_2_Pb_4_I_12_·2H_2_O under 1500 nm LCP and RCP laser excitation with the incident fluence of 10 mW, which was the largest value among the chiral lead‐based perovskite single crystals.

## 2 Results and Discussion

### 2.1 Crystal Structure Analysis

**Figure 1:**
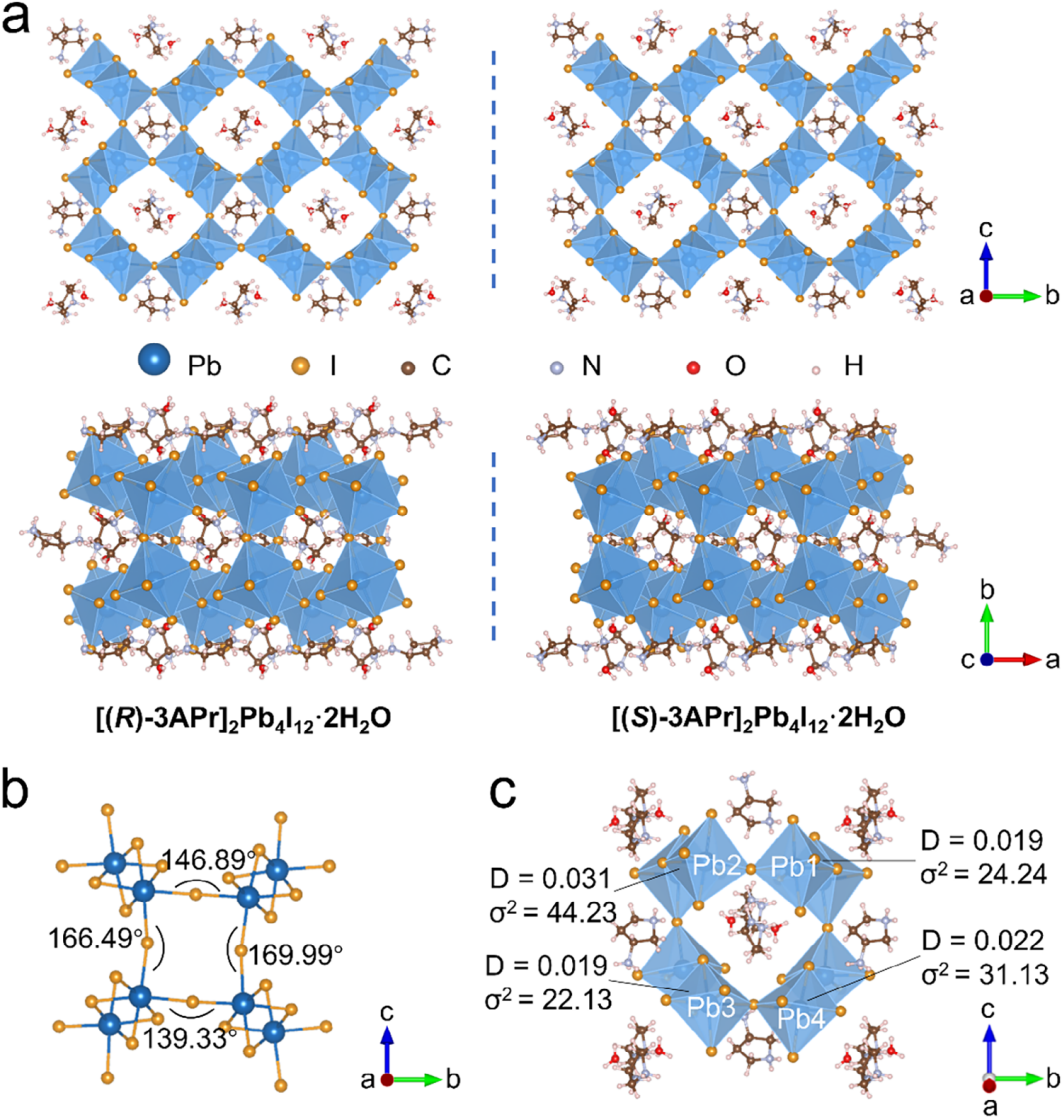
Crystal structure characterization of [(*R*)‐3APr]_2_Pb_4_I_12_·2H_2_O and [(*S*)‐3APr]_2_Pb_4_I_12_·2H_2_O perovskite single crystals. a) A schematic illustration of the chiral 3D perovskite structure of [(*R*)‐3APr]_2_Pb_4_I_12_·2H_2_O and [(*S*)‐3APr]_2_Pb_4_I_12_·2H_2_O perovskites. b) Inorganic frameworks showing the selected Pb─I─Pb bond angles of [(*R*)‐3APr]_2_Pb_4_I_12_·2H_2_O. c) Bond length distortion index (D) and the bond angle variance (σ^2^) in individual [PbI_6_] octahedron of [(*R*)‐3APr]_2_Pb_4_I_12_·2H_2_O.

Traditional 3D perovskites (AMX_3_) are constrained by the Goldschmidt tolerance factor range required for structural stability,^[^ ^39^ ^]^ making it difficult to directly incorporate large chiral cations into the perovskite lattice. By replacing PbX_6_^4−^ octahedra with Pb_2_X_10_^6−^ dimers composed of edge‐sharing octahedra as the fundamental building blocks enables effective expansion of the perovskite lattice.^[^ ^40^ ^]^ This structural regulation creates enlarged cavities capable of accommodating either two monovalent cations or one divalent cation, corresponding to the chemical formulas A_2_M_2_X_6_ and A'M_2_X_6_.^[^ ^41^ ^]^ However, within the A_2_M_2_X_6_ cavity, the significant electrostatic repulsion between the two positively charged monovalent cations renders this configuration energetically unfavorable, thereby increasing the synthesis difficulty. In contrast, asymmetric divalent cations offer a viable pathway for synthesizing A'M_2_X_6_‐type perovskites. Li et al. proposed a new tolerance factor rule to guide the design of 3D perovskite A'Pb_2_Br_6_.^[^ ^29^ ^]^ The size and shape of the diammonium cations are governed by the geometric constraints of the perovskite framework cavity. Bulk single crystals of [(*R*/*S*)‐3APr]_2_Pb_4_I_12_·2H_2_O (Figure S1, Supporting Information) were synthesized by reacting approximately stoichiometric amounts of (*R*/*S*)‐3APrCl_2_ with PbO_2_ in low‐concentration aqueous hydroiodic acid solution, employing a slight excess of PbO_2_ to prevent the formation of other phases. The chemical composition of [(*R*)‐3APr]_2_Pb_4_I_12_·2H_2_O was first investigated using X‐ray photoelectron spectroscopy (XPS). As shown in Figure S2 (Supporting Information), the wide XPS exhibits Pb, C, N, I and O peaks. The structure of the [(*R*/*S*)‐3APr]_2_Pb_4_I_12_·2H_2_O crystal was then analyzed by single crystal X‐ray diffraction (SC‐XRD) analysis. Both [(*R*)‐3APr]_2_Pb_4_I_12_·2H_2_O and [(*S*)‐3APr]_2_Pb_4_I_12_·2H_2_O crystallize in the same *P*2_1_ Sohncke space group, with almost identical cell parameters shown in Table S1 (Supporting Information). The powder X‐ray diffraction (PXRD) patterns of [(*R*/*S*)‐3APr]_2_Pb_4_I_12_·2H_2_O crystals are in good agreement with the simulated results, indicating their phase purity (Figure S3, Supporting Information). As shown in **Figure** **1**a, [(*R*/*S*)‐3APr]_2_Pb_4_I_12_·2H_2_O exhibit a distinct mirror symmetry framework structure. Chiral organic cations and H_2_O molecules located in the 3D framework cavities. Half of the framework cavities contained H_2_O molecules that formed N─H···O hydrogen bonds with the outer hydrogen atoms of pyrrole rings in adjacent chiral cations. Two octahedra are edge‐shared to form a dimer, which subsequently interconnects with adjacent dimers through corner‐sharing connectivity to form 3D network (Figure 1b). By treating the edge‐sharing octahedral pairs as rigid building blocks, only the Pb─I─Pb dihedral angles propagating along the corner‐sharing axis were considered. The average Pb─I─Pb angle of the 3D [PbI_6_] inorganic skeleton were calculated to be 155.68°, exhibiting pronounced inter‐octahedral distortions accompanied by substantial lattice deformation throughout the 3D perovskite framework. Owing to the heavily influence of the distortion level of individual [PbI_6_] octahedron on optoelectronic properties, the bond length distortion index (D) and the bond angle variance (σ^2^) of [(*R*)‐3APr]_2_Pb_4_I_12_·2H_2_O were calculated using following equations:

(1) $$D\;=\left(\frac{1}{6}\right)\sum_{i=1}^{6}\frac{\left|d_{i}-d_{\mathrm{av}}\right|}{d_{\mathrm{av}}}$$

(2) $$\sigma^{2}=\sum_{i=1}^{12}{\left(\theta_{i}-90\right)}^{2}/11$$

where d_i_ is the individual Pb─I bond length, d_av_ is the average Pb─I bond length, and θ_i_ is the individual I─Pb─I angle. As shown in Figure 1c, the pronounced inter‐octahedral distortion probably is attributed to the enhanced octahedral tilting effect arising from the asymmetric spatial configuration of NH_3_^+^ moieties within the chiral aromatic diammonium cations. The X‐ray diffraction (XRD) patterns of [(*R*)‐3APr]_2_Pb_4_I_12_·2H_2_O single crystal after 30‐days air exposure did not show any obvious new peaks, demonstrating its long‐term structural stability in the ambient environment (Figure S4, Supporting Information).

### 2.2 Optical and Electronic Properties

**Figure 2:**
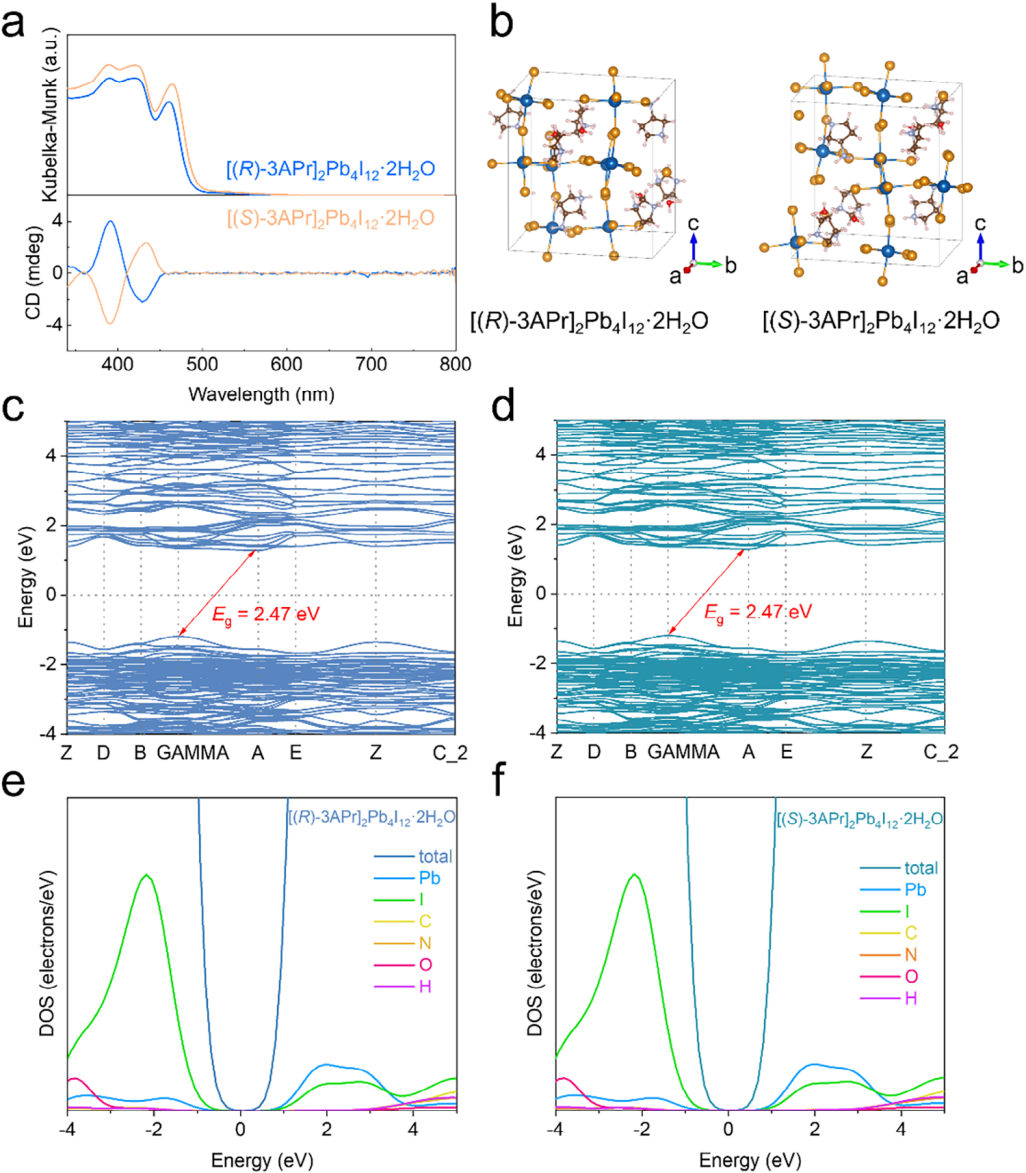
Optical properties and intrinsic energy band structure. a) The absorbance spectrum and CD spectra of [(*R*)‐3APr]_2_Pb_4_I_12_·2H_2_O and [(*S*)‐3APr]_2_Pb_4_I_12_·2H_2_O perovskites. b) The primitive cells of [(*R*)‐3APr]_2_Pb_4_I_12_·2H_2_O and [(*S*)‐3APr]_2_Pb_4_I_12_·2H_2_O perovskites used for DFT band structure calculations. Calculated electronic band structures of c) [(*R*)‐3APr]_2_Pb_4_I_12_·2H_2_O and d) [(*S*)‐3APr]_2_Pb_4_I_12_·2H_2_O perovskites. Calculated PDOS of e) [(*R*)‐3APr]_2_Pb_4_I_12_·2H_2_O and (f) [(*S*)‐3APr]_2_Pb_4_I_12_·2H_2_O perovskites.

To further investigate optical properties of [(*R*/*S*)‐3APr]_2_Pb_4_I_12_·2H_2_O crystal, UV–vis diffusive reflectance spectrum and CD spectra. As shown in **Figure** **2**a, the absorption spectroscopy of [(*R*/*S*)‐3APr]_2_Pb_4_I_12_·2H_2_O present an absorption onset at ≈2.47 eV, which is in good accordance with the light‐yellow appearance of the crystals. The CD spectra were also conducted to study the optical activity of [(*R*/*S*)‐3APr]_2_Pb_4_I_12_·2H_2_O. Mirrored CD signals near the absorption bands of [(*R*/*S*)‐3APr]_2_Pb_4_I_12_·2H_2_O are observed, which is a typical Cotton effects. This result indicates that the chirality was successfully transferred from the chiral organic ligands to the inorganic skeletons. The maximum *g*_CD_ value for [(*R*)‐3APr]_2_Pb_4_I_12_·2H_2_O perovskite was estimated as ≈0.002 (Figure S5, Supporting Information).

Density functional theory (DFT) calculations were performed to investigate the electronic band structures of the experimentally obtained structures. The energy band structure of the [(*R*/*S*)‐3APr]_2_Pb_4_I_12_·2H_2_O primitive cells (Figure 2b) was calculated by using GGA + PBE (generalized gradient approximation in the Perdew‐Burke‐Ernzerhof form) method neglecting the spin‐orbit coupling effect. [(*R*/*S*)‐3APr]_2_Pb_4_I_12_·2H_2_O exhibits indirect bandgap characteristic (Figure 2c,d), which can explain our failure in the photoluminescence measurements of the crystals. The valence band maximum (VBM) appears at the GAMMA point while the conduction band minimum (CBM) lies at the A point. The organic divalent cations indirectly and directly affect the valence and conduction bands of the perovskite, respectively.^[^ ^40^ ^]^ As shown in Figure 2c–f and corroborating structural data (Figure 2b), the indirect bandgap (*E*_g_ = 2.47 eV) in [(*R*/*S*)‐3APr]_2_Pb_4_I_12_·2H_2_O primarily originates from spontaneous symmetry breaking induced by the chiral organic cations [(*R*/*S*)‐3APr]^2+^. While the inorganic Pb–I sublattice fundamentally dictates the electronic band edges—evident in the dominance of Pb‐6p and I‐5p orbitals near the valence/conduction bands per partial density of states (PDOS) (Figure 2e,f)—the non‐centrosymmetric packing of chiral organic cations (Figure 2b) disrupts both the translational symmetry and inversion symmetry, folding the Brillouin zone and decoupling VBM/CBM positions. The CD spectra (Figure 2a) confirm enantiomeric asymmetry, validating this symmetry‐imposed constraint on band dispersion. The calculated bandgaps (2.47 eV) for [(*R*/*S*)‐3APr]_2_Pb_4_I_12_·2H_2_O is approach to the optical bandgap (2.52 eV) estimated through the Tauc plot (Figure S6, Supporting Information).

To further demonstrate the carrier transport characteristic of the prepared crystals, the effective masses of electrons and holes along three directions, N(011), N($01\bar{1}$), and [100] are calculated according to the following equation:

(3) $$\frac{1}{m^{*}}\;=\;\frac{1}{\hbar }\frac{d^{2}E\left(k\right)}{d^{2}k}$$

where N(hkl) represents the normal vector to (hkl), m* is the effective mass, *E*(*k*) and *k* represent the energy of a band and the wave vector, respectively. The first direction is the out‐of‐plane direction as confirmed by the XRD (Figure S7, Supporting Information), while the other two is the in‐plane directions. As shown in Table S2 (Supporting Information), the hole effective masses along the three crystallographic directions N(011), N($01\bar{1}$), and [100] are determined to be 0.479 *m*_0_, 0.479 *m*_0_, and 0.660 *m*_0_, respectively (where *m*_0_ represents the free electron mass), demonstrating nearly isotropic 3D charge transport characteristics in the crystal. The [PbI_6_]^4−^ octahedra are connected via an edge‐sharing mode along [100], while a combination of corner‐sharing and edge‐sharing connectivity are observed in the other two crystallographic directions. The hole effective mass along [100] is slightly higher than those in the other two directions, which is in agreement with the charge transport restriction within the edge‐sharing plane.^[^ ^29^, ^42^ ^]^ Since the edge‐sharing structural motif generally yields weaker band dispersion compared to the corner‐sharing configuration,^[^ ^41^, ^43^, ^44^, ^45^ ^]^ the electron effective mass along [100] is consequently significantly higher than those along the other two directions. The PDOS around the Fermi level analysis (Figure 2e,f) show that the octahedral framework in the perovskite [(*R*/*S*)‐3APr]_2_Pb_4_I_12_·2H_2_O lattice plays a dominant role to the energy bandgap. The isosurface plots of the wave functions for the VBM and CBM further confirm that the charge density is predominantly localized on the [PbI_6_] octahedra (Figure S8, Supporting Information). The isosurface plot of the VBM wave function exhibits a 3D electronic delocalization characteristic, while the carrier continuity along the *c*‐axis direction in the CBM appears relatively weak. This observation further highlights the crucial role of the inorganic framework in governing the electronic properties of the perovskite structure, where the [PbI_6_] octahedra serve as the primary medium for charge carrier generation and transport. The carrier mobility of the [(*R*)‐3APr]_2_Pb_4_I_12_·2H_2_O perovskite single crystal in three orientations lies within the same order of magnitude (Figure S9, Supporting Information), approaching the mobility level of 3D perovskite MAPbI_3_ single crystals.^[^ ^46^ ^]^

### 2.3 CPL Photodetection

**Figure 3:**
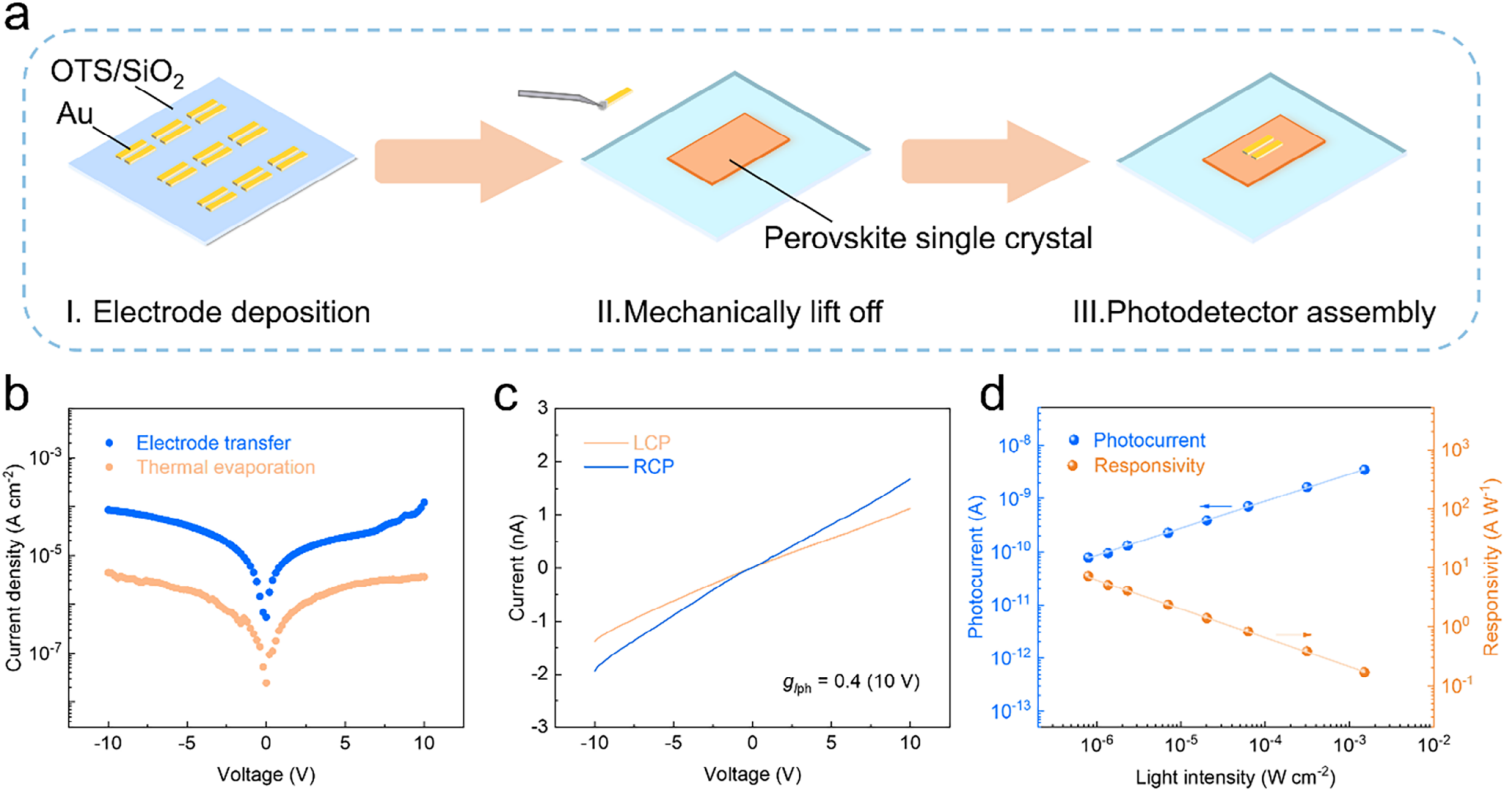
Dedicated device fabrication methodology for CPL photodetector. a) The schematic fabrication processes for the [(*R*)‐3APr]_2_Pb_4_I_12_·2H_2_O‐based photodetector. b) The current density‐voltage (*J*‐*V*) curves of photodetector fabricated by thermal evaporation and electrode transfer method. c) The current‐voltage (*I*–*V*) curves of the device fabricated by electrode transfer strategy under LCP and RCP light illumination at the wavelength of 435 nm. d) Photocurrent and corresponding responsivity for the photodetector fabricated by electrode transfer method.

Firstly, devices are fabricated by thermally evaporating gold (Au) electrodes on the crystal surface through a specific metal shadow mask. However, the crystal undergoes a degradation from a transparent, crack‐free state to an opaque bulk solid exhibiting inhomogeneous coloration, as shown in the photographs of pristine single crystal and thermal evaporation‐treated single crystal (Figure S10a, Supporting Information). Furthermore, the chemical composition information of single crystal was then investigated by XPS measurements. It is noteworthy that the high vacuum and elevated temperatures during the vacuum evaporation process cause crystal degradation and dehydration (Figure S10b, Supporting Information), which is highly detrimental to device performance. Thermogravimetric analysis (TGA) substantiates the inherent instability of lattice‐bound water molecules in perovskite frameworks, demonstrating their propensity for rapid thermal desorption under ambient conditions (Figure S10c, Supporting Information). To accurately assess the optoelectronic performance of photodetectors based on this chiral 3D perovskite single crystal, a dedicated device fabrication methodology was developed. As shown in **Figure** **3**a, the Au electrodes were thermally evaporated onto octadecyltrichlorosilane (OTS)‐functionalized SiO_2_/Si substrates using high‐precision shadow masks for electrode patterning. The reduced surface energy of the OTS‐modified interface facilitated complete probe‐assisted transfer of the pre‐patterned Au microelectrodes with maintained interfacial integrity. Following the transfer of pre‐patterned Au microelectrodes onto the perovskite single‐crystal surface, photodetector with a photoconductive device structure of Au/perovskite/Au was successfully constructed and subsequently subjected to electrical characterization. As delineated in Figure 3b, the photocurrent density of perovskite photodetectors constructed via probe‐assisted transfer electrode methodology is one order of magnitude higher than that of the device fabricated by thermal evaporation versus under identical monochromatic illumination (435 nm, 1.5 mW cm^−2^). Therefore, the probe‐assisted electrode‐transfer strategy demonstrates its effectiveness in evaluating photo response characteristics, thereby providing a robust platform for accurately assessing the performance of optoelectronic devices based on perovskite single crystals with inherent thermal instability.

The exceptional CPL absorption of [(*R*)‐3APr]_2_Pb_4_I_12_·2H_2_O perovskites can be harnessed for CPL photodetection. A 435 nm CPL was employed to investigate the CPL detection performance of [(*R*)‐3APr]_2_Pb_4_I_12_·2H_2_O crystal‐based device. The *I*−*V* curves of [(*R*)‐3APr]_2_Pb_4_I_12_·2H_2_O perovskite device under RCP and LCP light illumination are presented in Figure 3c. An obvious unidentical photo response can be observed in the device, demonstrating the prominent capability to differentiate between RCP and LCP light. At the same light intensity, the photocurrent generated under RCP light illumination exceeds that under LCP light illumination. To quantify the distinguishability of CPL for [(*R*)‐3APr]_2_Pb_4_I_12_·2H_2_O single crystal, the anisotropy factor for photocurrent (*g_I_*_ph_) is defined using the equation: *g_I_*_ph_ = 2(*I*_L_ − *I*_R_) / (*I*_L_ + *I*_R_), where *I*_L_ and *I*_R_ represent the photocurrent under LCP and RCP light illumination, respectively. The calculation yielded a *g_I_*_ph_ of 0.4, comparable to the state‐of‐the‐art chiral perovskite‐based detectors.^[^ ^47^ ^]^ The high *g_I_*_ph_ can be attributed to the strong chiral‐induced spin selectivity (CISS) effect in the chiral perovskite single crystals.^[^ ^48^, ^49^ ^]^

Maintaining linear photoresponse dependence on illumination intensity proves critical in practical photodetector operations.^[^ ^50^ ^]^ Photoelectric measurements under varied optical power densities were taken on [(*R*)‐3APr]_2_Pb_4_I_12_·2H_2_O bulk single crystal‐based device. The light intensity‐dependent photocurrent and responsivity displayed in Figure 3d. The device demonstrates linear optical‐electrical response characteristics within an illumination intensity range of 7.9 × 10^−4^–1.5 mW cm^−2^, exhibiting a corresponding linear dynamic range (LDR) > 65 dB. Additionally, the responsivity (*R*) stands as a pivotal figure‐of‐merit for evaluating the performance metrics of photodetectors, which can be determined through the equation *R* = *I*_ph_/*PA*, where *I*_ph_ is photocurrent, *P* is the incident light power density, *A* is effective illumination area of the photodetector. The peak value of responsivity was evaluated to be 7.2 A W^−1^. Specific detectivity (D*) is another important metric, the highest detectivity of 9.4 × 10^12^ Jones (10 V bias), calculated according to the reported method,^[^ ^26^ ^]^ represents record‐high sensitivity for perovskite photodetectors. This is on par with that of their 3D counterparts‐based CPL photodetectors^[^ ^26^ ^]^ and comparable commercially available silicon photodiodes (10^13^ Jones).^[^ ^51^, ^52^ ^]^

### 2.4 NLO Properties

**Figure 4:**
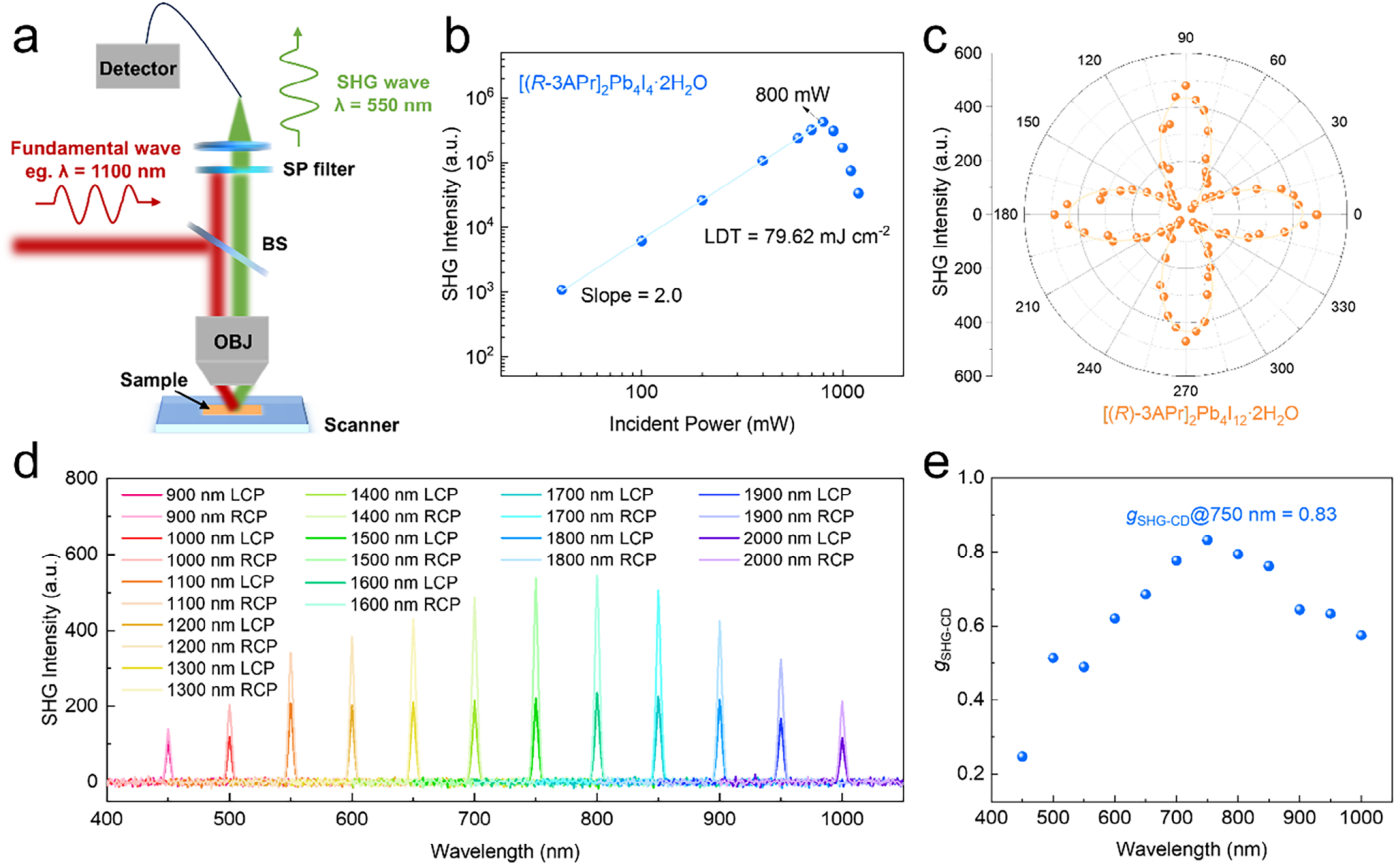
Circularly polarized‐dependent SHG effect. a) Schematic of the NLO measurement. b) Logarithmic plot of the SHG intensity for [(*R*)‐3APr]_2_Pb_4_I_12_·2H_2_O single crystal with the incident power density. c) Polarization dependence of SHG intensity as a function of the linear polarization angles. The orange line is the nonlinear fitting of data points. d) SHG intensity of [(*R*)‐3APr]_2_Pb_4_I_12_·2H_2_O single crystal under LCP and RCP laser pump. The pump wavelength extended from 900 to 2000 nm with 100 nm intervals. e) Wavelength‐dependence of anisotropy factors of [(*R*)‐3APr]_2_Pb_4_I_12_·2H_2_O single crystal.

As a pivotal branch of NLO processes, SHG represents a second‐order frequency‐doubling response arising from intense light‐matter interactions. Owing to different linear absorption rates (different refractive indices) of chiral materials for LCP and RCP light, different SHG signal efficiency can be obtained when circularly polarized lasers pass through chiral perovskite single crystals at different velocities, offering exciting opportunities for CPL sensing. To study the second‐order NLO properties for the chiral 3D perovskite single crystals, a home‐built confocal optical microscope equipped with a Chameleon Ti: Sapphire lasers (≈ 100 fs, 80 MHz) was used as the pump, and the SHG signals were collected in reflection configuration. The schematic illustration of the NLO experimental setup is depicted in **Figure** **4**a. The wavelength‐dependent SHG measurements were performed through wavelength‐tunable excitation ranging from 970 to 1210 nm with 30 nm intervals, while constant incident fluence (10 mW) was maintained. As shown in Figure S11 (Supporting Information), the SHG spectra obtained from the [(*R*)‐3APr]_2_Pb_4_I_12_·2H_2_O single crystal demonstrate a pronounced SHG signal when excited at various wavelengths in the NIR region. Superior photostability serves as a critical prerequisite for practical implementation of NLO crystals. Therefore, we conducted comprehensive studies on the laser damage threshold (LDT) and NLO response stability of [(*R*)‐3APr]_2_Pb_4_I_12_·2H_2_O crystal exposed to high‐repetition‐rate femtosecond laser pulses. The quadratic dependence of SHG intensity on the incident laser power unequivocally confirms the two‐photon excitation mechanism governing the SHG process. This characteristic nonlinear response persists up to an incident power of 800 mW (corresponding to a laser spot diameter of 4 µm). Beyond this threshold power density (Figure 4b), [(*R*)‐3APr]_2_Pb_4_I_12_·2H_2_O crystal exhibits significant SHG intensity degradation. The measured LDT of 79.62 mJ cm^−2^ for [(*R*)‐3APr]_2_Pb_4_I_12_·2H_2_O substantially exceeds values reported for most chiral perovskites, including (*R*‐MPEA)_1.5_PbBr_3.5_(DMSO)_0.5_ nanowires (≈ 0.52 mJ cm^−2^), (*S*‐2‐MPD)PbBr_3_ (≈ 2.84 mJ cm^−2^), and (*R*‐MBA)CuBr_2_ microplates (1.7 mJ cm^−2^). These results demonstrate exceptional photostability in the synthesized chiral perovskite [(*R*)‐3APr]_2_Pb_4_I_12_·2H_2_O single crystal under intense laser excitation.

### 2.5 Polarized‐Dependent SHG Effect

As illustrated in Figure 4c, [(*R*)‐3APr]_2_Pb_4_I_12_·2H_2_O exhibits characteristic dipole‐like SHG response profiles at 1500 nm excitation, which can be well‐fitted with a cos^4^θ function. The corresponding degree of polarization (*ρ*) can be quantitatively determined using the following expression:

(4) $$\rho =\frac{I_{\max}-I_{\min}}{I_{\max}+I_{\min}}$$

where *I*_max_ and *I*_min_ represent the maximum and minimum SHG intensities, respectively. Quantitative analysis yields *ρ* of 88.5% for [(*R*)‐3APr]_2_Pb_4_I_12_·2H_2_O. The result demonstrates that [(*R*)‐3APr]_2_Pb_4_I_12_·2H_2_O exhibits superior optical anisotropy under linearly polarized pumping conditions. The polarization angle corresponding to SHG maximum theoretically aligns with the crystalline dipole axis. In [(*R*)‐3APr]_2_Pb_4_I_12_·2H_2_O, the emergence of this dipole axis originates from the incorporation of chiral organic cations, which facilitate substantial molecular rotational freedom. This structural characteristic induces inversion symmetry breaking, thereby generating long‐range polar ordering within the crystal lattice.

To further investigate the circularly polarized SHG characteristics of this chiral perovskite single crystal, circularly polarized‐sensitive SHG measurements were carried out to explore its potential application in the field of CPL detection in the NIR regime. Thus, different SHG intensity were observed in [(*R*)‐3APr]_2_Pb_4_I_12_·2H_2_O single crystal when pumped under LCP and RCP pump laser upon 900–2000 nm excitation (Figure 4d). [(*R*)‐3APr]_2_Pb_4_I_12_·2H_2_O exhibited pronounced different SHG intensity under LCP and RCP laser pump at the wavelength of 750 nm. However, this discrepancy becomes marginal at 450 nm and 1000 nm wavelengths, demonstrating wavelength‐dependent circularly polarized sensitivity in NIR. To quantitatively evaluate the circular polarization sensitivity of SHG, an anisotropy factor (*g*_SHG‑CD_) can be defined as *g*_SHG‑CD_ = 2(*I*_RCP_ − *I*_LCP_) / (*I*_LCP_ + *I*_RCP_) according to the previous report, where *I*_RCP_ and *I*_LCP_ represent the measured SHG intensities under LCP and RCP irradiation, respectively. As depicted in Figure 4e, a highest *g*_SHG‑CD_ value of 0.83 was achieved in [(*R*)‐3APr]_2_Pb_4_I_12_·2H_2_O single crystal upon 1500 nm excitation, which is surpasses that of Pb‐based perovskite ((*R*/*S*)‐3‐aminopiperidine)PbI_4_ crystal (0.21, 1064 nm)^[^ ^53^ ^]^ and (*R*‐ MPEA)_1.5_PbBr_3.5_(DMSO)_0.5_ nanowire (0.74, 850 nm).^[^ ^20^ ^]^ Moreover, the *g*_SHG‑CD_ can be tuned by the pumped wavelength, confirming pronounced circular polarization sensitivity in the NIR region.

## 3 Conclusion

In conclusion, we report a novel 3D chiral lead halide perovskite single crystal [(*R*/*S*)‐3APr]_2_Pb_4_I_12_·2H_2_O, which contains a unique 3D lead‐iodine framework of both corner‐sharing and edge‐sharing [PbI_6_] octahedra. The chiral 3D perovskite‐based photodetectors were fabricated by an electrode‐transfer methodology, exhibiting excellent detection performance with a circular‐polarization anisotropy factor of 0.4. This device assembly strategy offers an efficient approach for the device fabrication based on materials with inherent thermal instability. Owing to excellent NLO properties, [(*R*)‐3APr]_2_Pb_4_I_12_·2H_2_O single crystal exhibit remarkable distinguish ability for the LCP and RCP light in the NIR through different efficiencies of SHG signal. The *g*_SHG‐CD_ of [(*R*)‐3APr]_2_Pb_4_I_12_·2H_2_O single crystal is as high as 0.83 under 1500 nm excitation with the intensity of 10 mW. This research provides a new perspective for exploring chiral 3D lead halide perovskites.

## Conflict of Interest

The authors declare no conflict of interest.

## Supporting information



Supporting Information

## Data Availability

The data that support the findings of this study are available from the corresponding author upon reasonable request.
