# Supplementary material for: Chiral 3D Perovskite Single Crystals Realized by Lattice Expansion
Source: Adv Sci (Weinh). 2025 Jul 1;12(37):e06902. doi: 10.1002/advs.202506902 (PMC12499401; doi:10.1002/advs.202506902)
Supplement: Supplementary file 1 — Supporting Information [file ADVS-12-e06902-s001.docx]

**Supporting Information**

**Chiral 3D Perovskite Bulk Single Crystal Realized by Lattice Expansion**

*Lin Wang, Wei Hao, Shen Chen, Jie Ren*, Hanying Li**

MOE Key Laboratory of Macromolecular Synthesis and Functionalization, International Research Center for X Polymers,

Department of Polymer Science and Engineering

Zhejiang University, Hangzhou 310027, China

*Corresponding authors. Email: [jie.ren@zju.edu.cn](mailto:jie.ren@zju.edu.cn) (J.R.), [hanying_li@zju.edu.cn](mailto:hanying_li@zju.edu.cn) (H.L.)

**1. Experimental Section**

***Materials***: Lead (II) oxide (PbO) (>99.0%), hydriodic acid (HI) solution (45 ωt% in H_2_O, stabilized with 1.5% hypophoaphoeous acid), hypophosphorous acid solution (H_3_PO_2_) (50 ωt% in H_2_O) were purchased from Aladdin (Shanghai, China). (3*S*)-(+)-3-Aminopyrrolidine dihydrochloride [(*S*)-3AprCl_2_] and (3*R*)-(-)-3-Aminopyrrolidine dihydrochloride [(*R*)-3AprCl_2_] were purchased from Bide Pharmatech Ltd (Shanghai, China). Gallium Indium alloy (Ga:In = 40:60 ωt%) was purchased from Aladdin (Shanghai, China). All the reagents were used without further purification.

***Synthesis***: [(*R*)- and (*S*)-3APr]_2_Pb_4_I_12_·2H_2_O bulk single crystals were grown by a temperature colling method. Specifically, PbO (0.47 g) was first dissolved in a mixture of hydroiodic acid (5 mL) and hypophosphorous acid solution (1 mL) at 90°C, to yield a pale-yellow solution. (*R*)-3AprCl_2_ (0.16 g) was added to the resulting transparent solution with continuous heating at 90°C until the precipitate was dissolved. Then, the solution was then kept in an oven and slowly cooled to room temperature with a cooling rate of 1°C/2 h. Many yellow bulk crystals started to grow after 6 days. The obtained crystals were filtered and dried on a filter paper for a further 30 min. Synthetic method for [(*S*)-3APr]_2_Pb_4_I_12_·2H_2_O SCs is similar.

Vacuum thermal evaporation of Device: Planar structured photodetectors were fabricated by depositing Cr (5 nm)/Au (50 nm) electrodes method on the surface of [(*R*)-3APr]_2_Pb_4_I_12_·2H_2_O single crystal via vacuum thermal evaporation. The channel length between neighboring electrodes were determined to be 30 μm, while the channel width is 220 μm.

Electrode-Transfer Fabrication of Device: Au source electrodes of 40 nm were vacuum-deposited via thermal evaporation on the octadecyltrichlorosilane (OTS)-modified silicon wafers through a shadow mask. Thereafter, the resulting electrodes were transferred to the surface of [(*R*)-3APr]_2_Pb_4_I_12_·2H_2_O single crystal with the aid of a probe dipped in Gallium Indium alloy. The completed devices were stored in a vacuum oven (< 1 mbar) for 1 h before performing electrical characterizations. The channel length between neighboring electrodes were determined to be 18 μm, while the channel width is 76 μm.

***Characterization***: Powder X-ray diffraction (XRD) patterns were performed by a Rigaku Ultima IV X-ray diffractometer in the 2𝜃 range 5°-40° with a step size of 0.02°. Optical diffuse reflectance measurements were measured by a Shimadzu UV-2600i UV-vis-NIR spectrophotometer equipped using BaSO_4_ as the reference standard. The transmission circular dichroism (CD) spectra were conducted using a JASCO J-1700 CD spectrometer with a KBr pellet background. Thermogravimetric Analysis (TGA) were carried out by a TA-Waters Discovery TGA55 thermal analyzer. X-ray photoelectron spectroscopy (XPS) was performed on a Shimadzu axis supra photoelectron spectrometer. Single-crystal structural data was performed on a Bruker D8 Venture diffractometer using Mo K𝛼 radiation (𝜆 = 0.71073). Crystal structures of [(*R*)- and (*S*)-3APr]_2_Pb_4_I_12_·2H_2_O were solved with the SHELXT structure solution program using intrinsic pphasing and refined with the SHELXL refinement package using Least Squares minimization.

***Nonlinear Optical Measurements***: A Chameleon Ti: Sapphire lasers (≈ 100 fs, 80 MHz) centered at 808 nm is used as the pump and fix the [(*R*)-3APr]_2_Pb_4_I_12_·2H_2_O bulk single crystal on the substrate for second harmonic generation (SHG) measurements. And the resulting signal was collected by the WITec alpha 300RS+ Raman system with a 50 × objective.

***Detector performance measurement***: The photoresponse characteristics of the photodetectors were conducted by using a Keithley 4200-SCS semiconductor analyzer. A monochromatic light beam with wavelength of 435 nm was generated by a xenon lamp (Microenerg, CME-TLSX300F) coupled with a monochromator. The output monochromatic beam then passed through a linear polarizer, a quarter-wave plate to obtain circularly polarized light (CPL), and was perpendicularly irradiated onto devices. The light intensity was calibrated using a light power meter (PM101A, Thorlabs). All measurements were performed in vacuum (≈ 10^–3^ mbar) at room temperature.

***Theoretical calculation details***: All theoretical calculations were carried out by employing the first-principles simulations based on density functional theory (DFT) within the Vienna Ab-initio Simulation Package (VASP)^[1,2]^. The projector augmented wave (PAW) method was used to describe the electron–ion interaction and the exchange-correlation between electrons was described by the generalized gradient approximation (GGA) in the Perdew-Burke-Ernzerhof (PBE) form^[3,4]^. A cutoff energy of 500 eV was used for the plane-wave basis set in all calculations. All the atoms in the model were relaxed until the force on each atom is below 0.01 eV/Å. The Brillouin-zone integrations were performed using a (4 × 2 × 2) *k*-mesh of the Monkhorst-Pack sampling scheme^[5]^ to obtain their electronic properties.

CCDC 2430452 and 2430458 contains the supplementary crystallographic data for this paper. These data can be obtained free of charge from The Cambridge Crystallographic Data Centre via www.ccdc.cam.ac.uk/data_request/cif.

**References**

[1] G. Kresse, J. Furthmüller, *Phys. Rev. B* **1996**, *54*, 11169–11186.

[2] G. Kresse, J. Furthmüller, *Comp. Mater. Sci.* **1996**, *6*, 15–50.

[3] J. P. Perdew, K. Burke, M. Ernzerhof, *Phys. Rev. Lett.* **1996**, *77*, 3865–3868.

[4] G. Kresse, D. Joubert, *Phys. Rev. B* **1999**, *59*, 1758–1775.

[5] H. J. Monkhorst, J. D. Pack, *Phys. Rev. B* **1976**, *13*, 5188–5192.

**Table S1.** Crystal data for [(*R*)-3APr]_2_Pb_4_I_12_·2H_2_O and [(*S*)-3APr]_2_Pb_4_I_12_·2H_2_O perovskites at ambient condition.

| Perovskite crystal | [(*R*)-3APr]_2_Pb_4_I_12_·2H_2_O | [(*S*)-3APr]_2_Pb_4_I_12_·2H_2_O |
| --- | --- | --- |
| Temperature (K) | 170 | 170 |
| Formula weight | 2563.9 | 2563.9 |
| V (Å^3^) | 2007.18(15) | 2005.68(13) |
| Crystal system | monoclinic | monoclinic |
| Space group | *P*2_1_ | *P*2_1_ |
| Cell parameters | a (Å) = 7.7223(4)  b (Å) = 16.0963(6)  c (Å) = 16.4749(7)  α (°) = 90  β (°) = 101.436(2)  γ (°) = 90 | a (Å) = 7.7223(3)  b (Å) = 16.0880(6)  c (Å) = 16.4689(6)  α (°) = 90  β (°) = 101.3980(10)  γ (°) = 90 |
| Z | 2 | 2 |
| R_1_ | 0.0177 | 0.0225 |

**Table S2.** Effective masses of [(*R*)-3APr]_2_Pb_4_I_12_·2H_2_O and [(*S*)-3APr]_2_Pb_4_I_12_·2H_2_O perovskites.

| Materials | Direction | *m*_e_ | *m*_h_ |
| --- | --- | --- | --- |
| [(*R*)-3APr]_2_Pb_4_I_12_·2H_2_O | N(011) | 0.445 | -0.479 |
|  | N($\text{01}\bar{\text{1}}$) | 0.445 | -0.479 |
|  | [100] | 3.733 | -0.660 |
| [(*S*)-3APr]_2_Pb_4_I_12_·2H_2_O | N(011) | 0.450 | -0.480 |
|  | N($\text{01}\bar{\text{1}}$) | 0.450 | -0.480 |
|  | [100] | 3.642 | -0.664 |


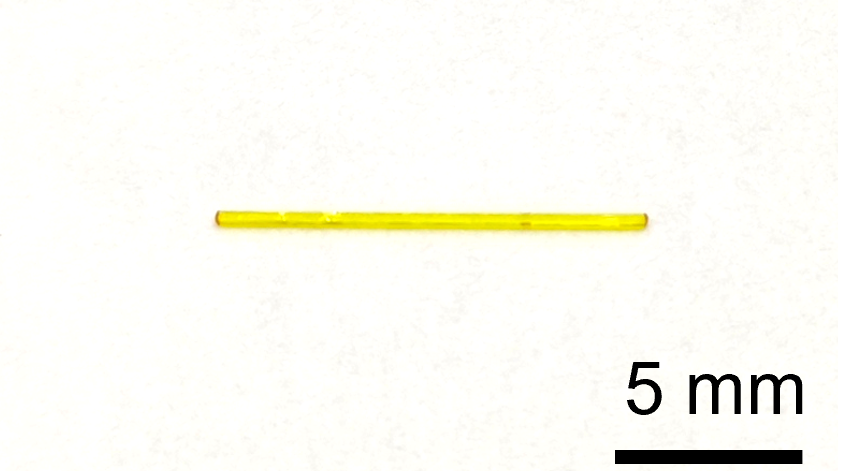


**Figure S1.** Photos of bulk [(*R*)-3APr]_2_Pb_4_I_12_·2H_2_O single crystal.


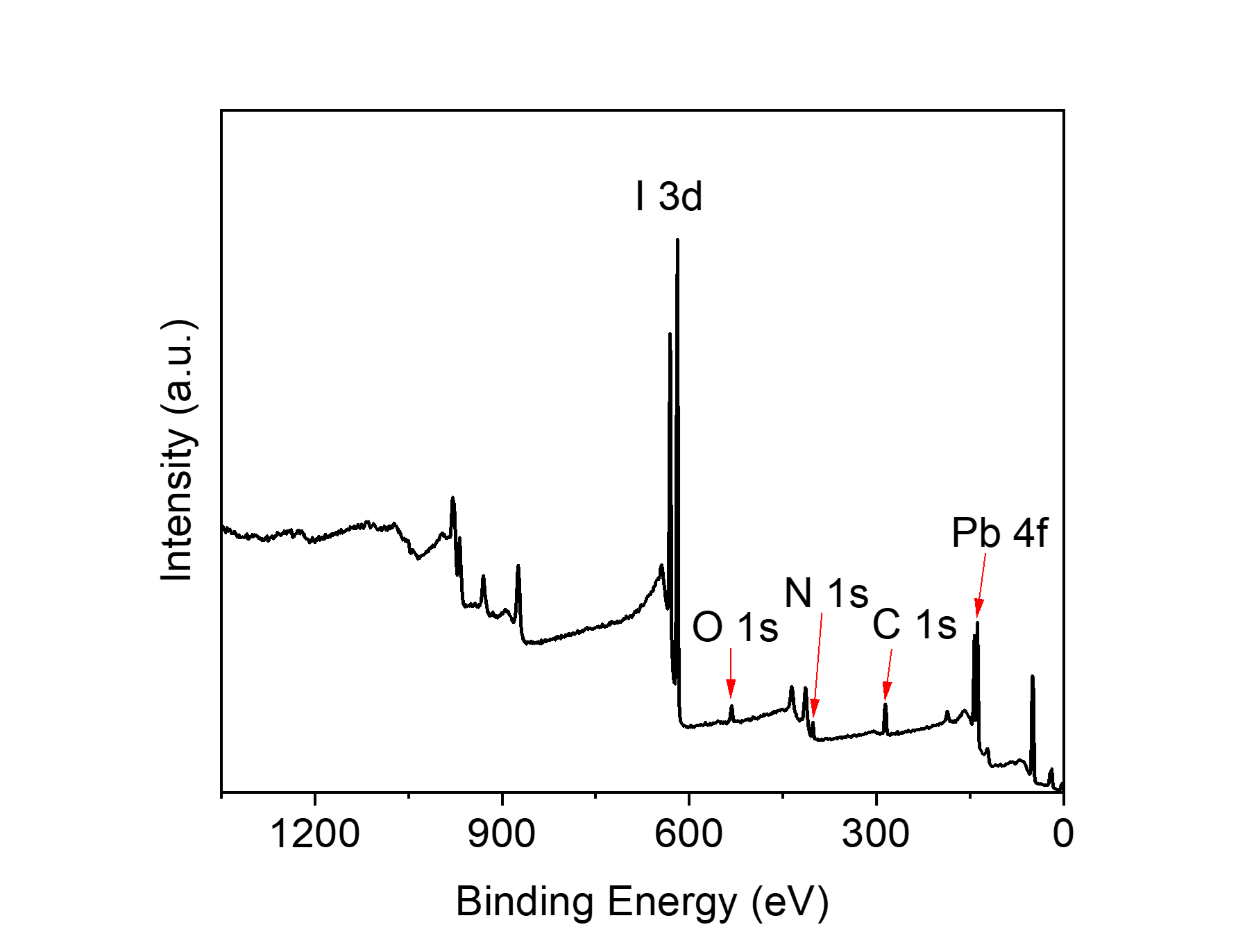


**Figure S2.** XPS for [(*R*)-3APr]_2_Pb_4_I_12_·2H_2_O single crystal.


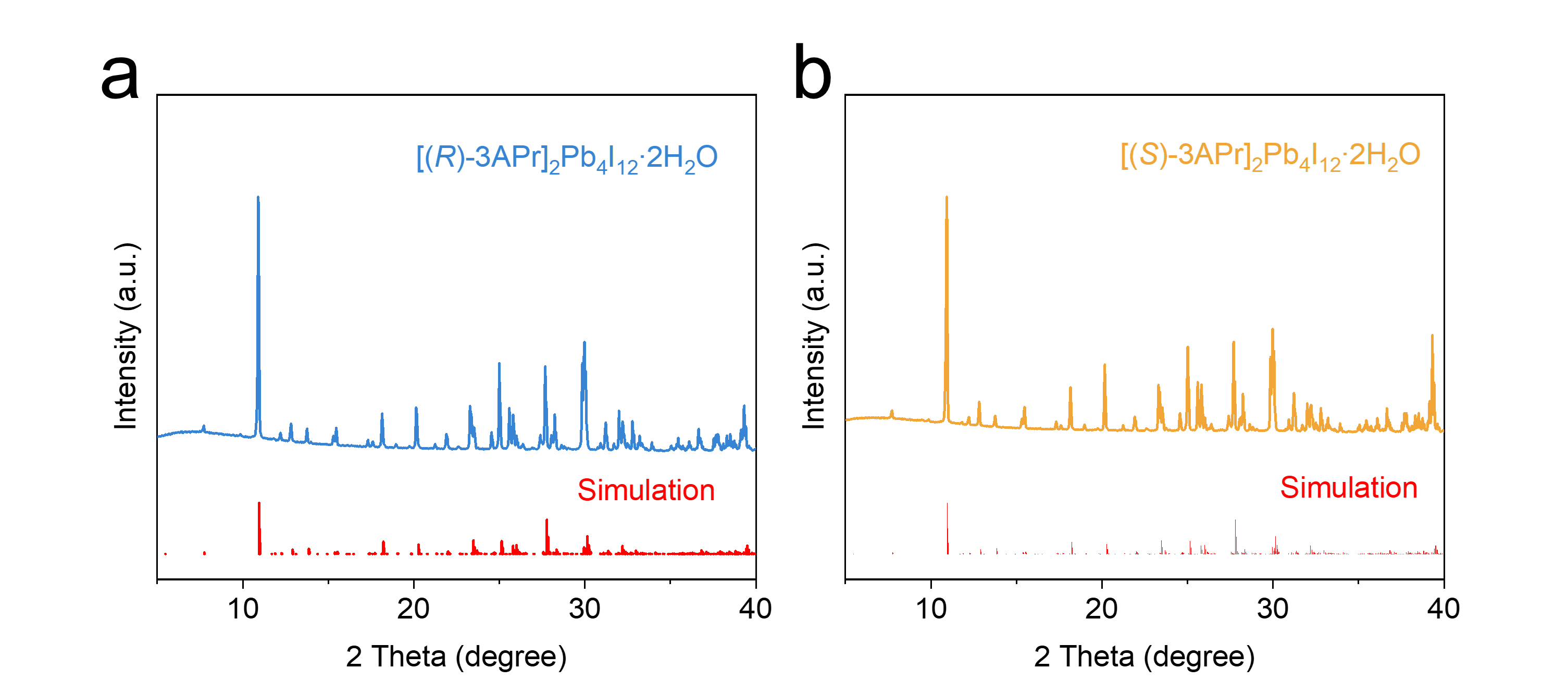


**Figure S3.** Experimental and simulated powder XRD profiles of (a) [(*R*)-3APr]_2_Pb_4_I_12_·2H_2_O and (b) [(*S*)-3APr]_2_Pb_4_I_12_·2H_2_O perovskites.


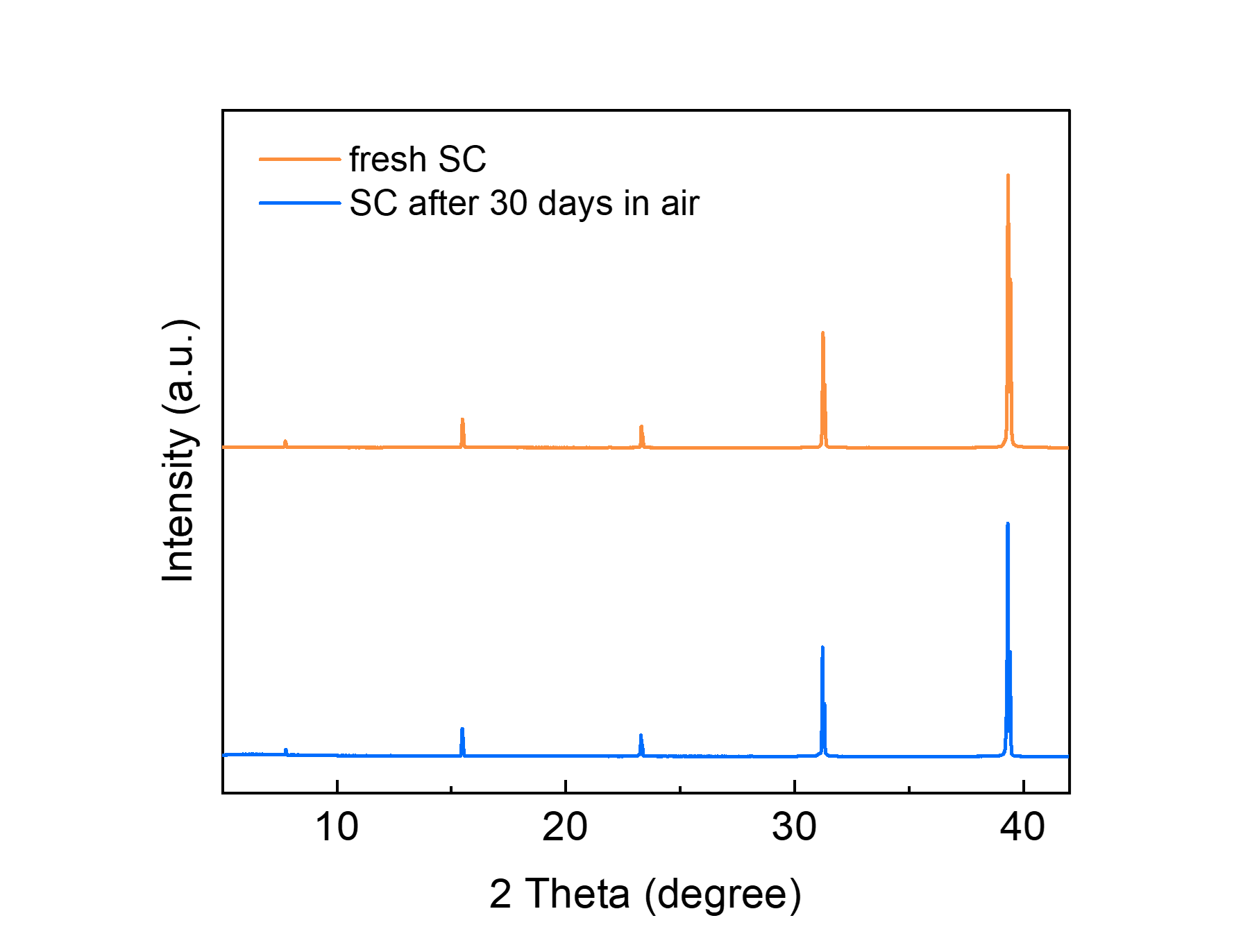


**Figure S4.** The XRD patterns of [(*R*)-3APr]_2_Pb_4_I_12_·2H_2_O single crystal before and after 30-day air exposure.


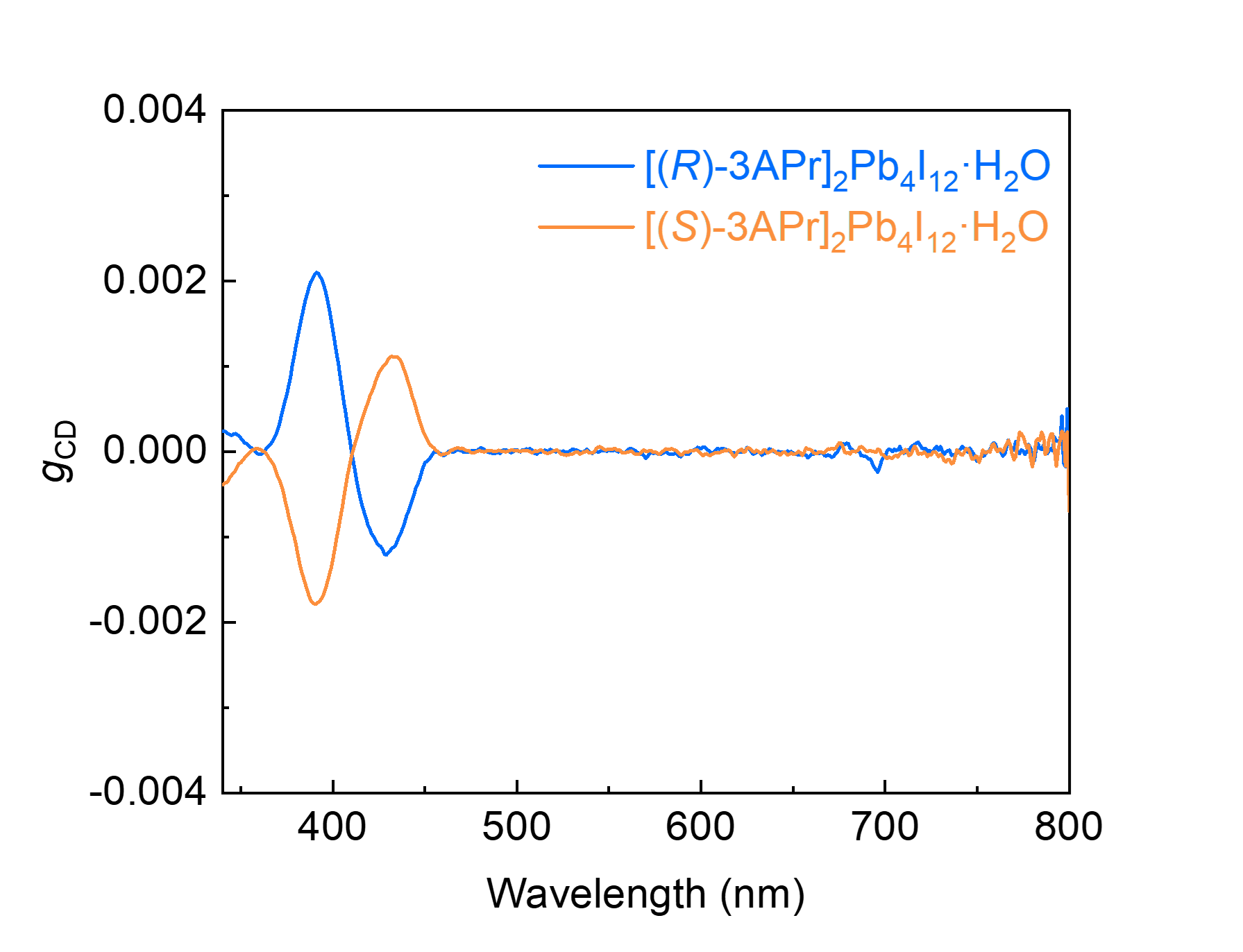


**Figure S5.** The anisotropy factor for CD (*g*_CD_) of [(*R*)-3APr]_2_Pb_4_I_12_·2H_2_O and [(*S*)-3APr]_2_Pb_4_I_12_·2H_2_O.


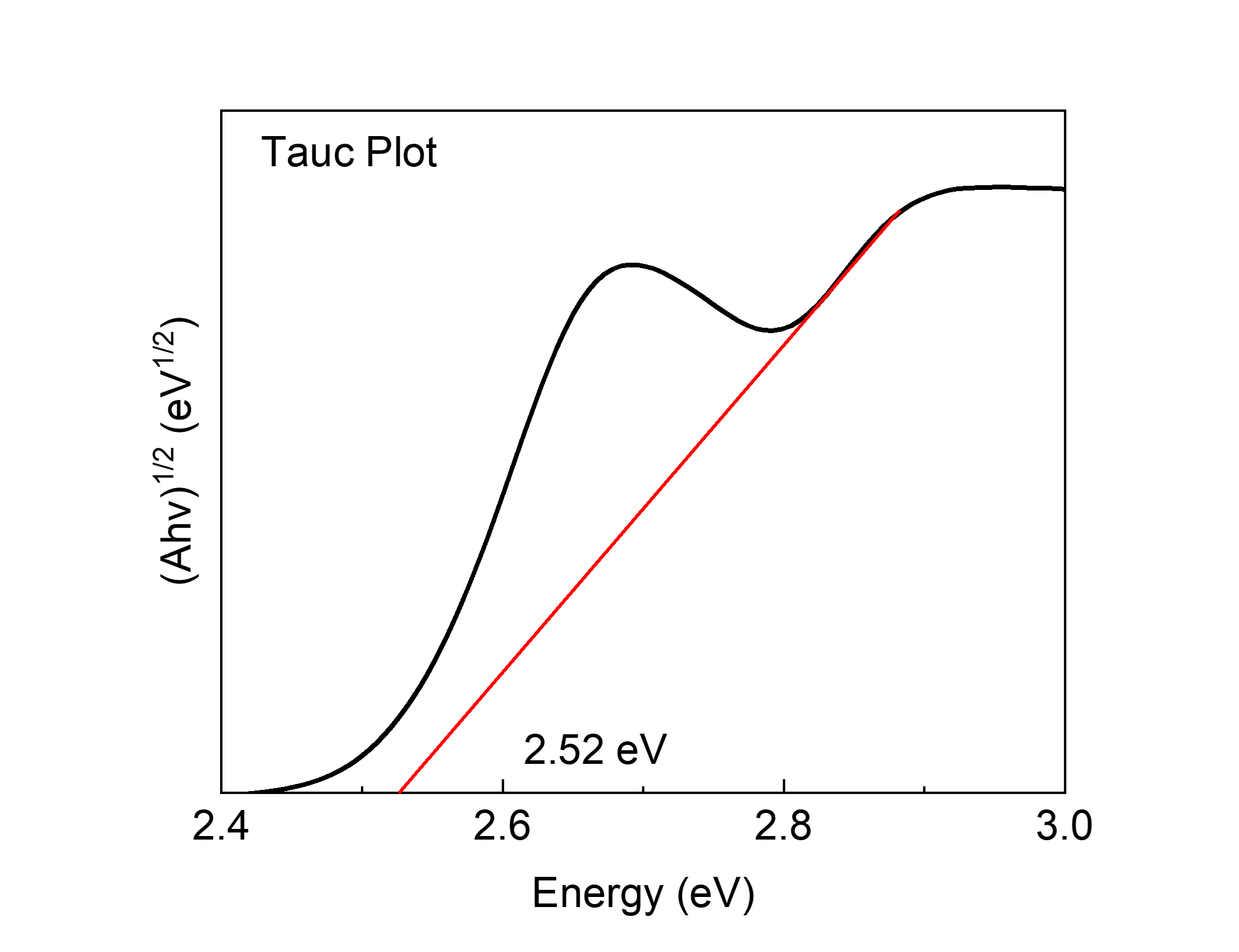


**Figure S6.** The corresponding Tauc plot of absorbance spectrum of [(*R*)-3APr]_2_Pb_4_I_12_·2H_2_O single crystal, showing a band gap 2.52 eV.


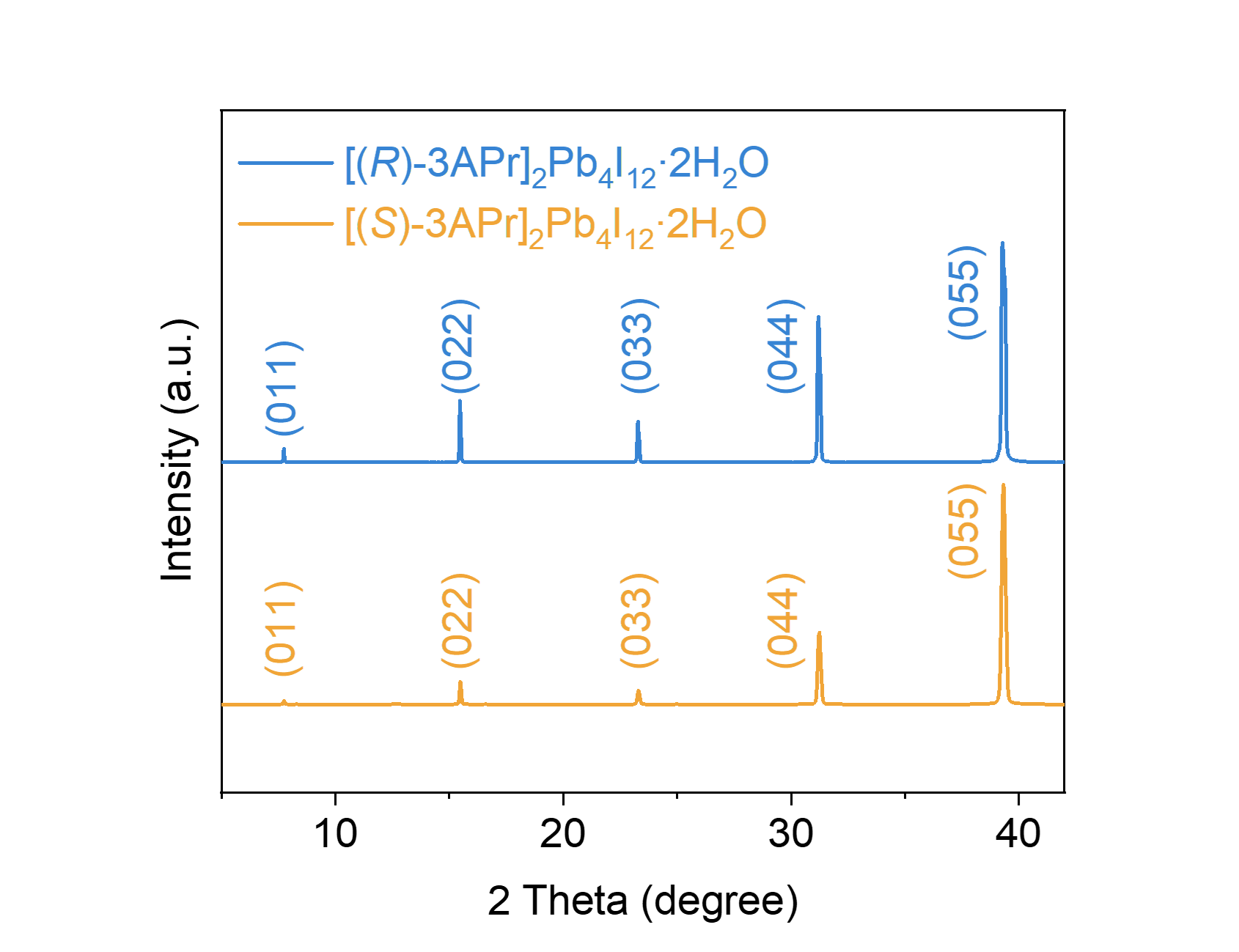


**Figure S7.** The XRD patterns of [(*R*)-3APr]PbI_4_ and [(*S*)-3APr]PbI_4_ single crystals.


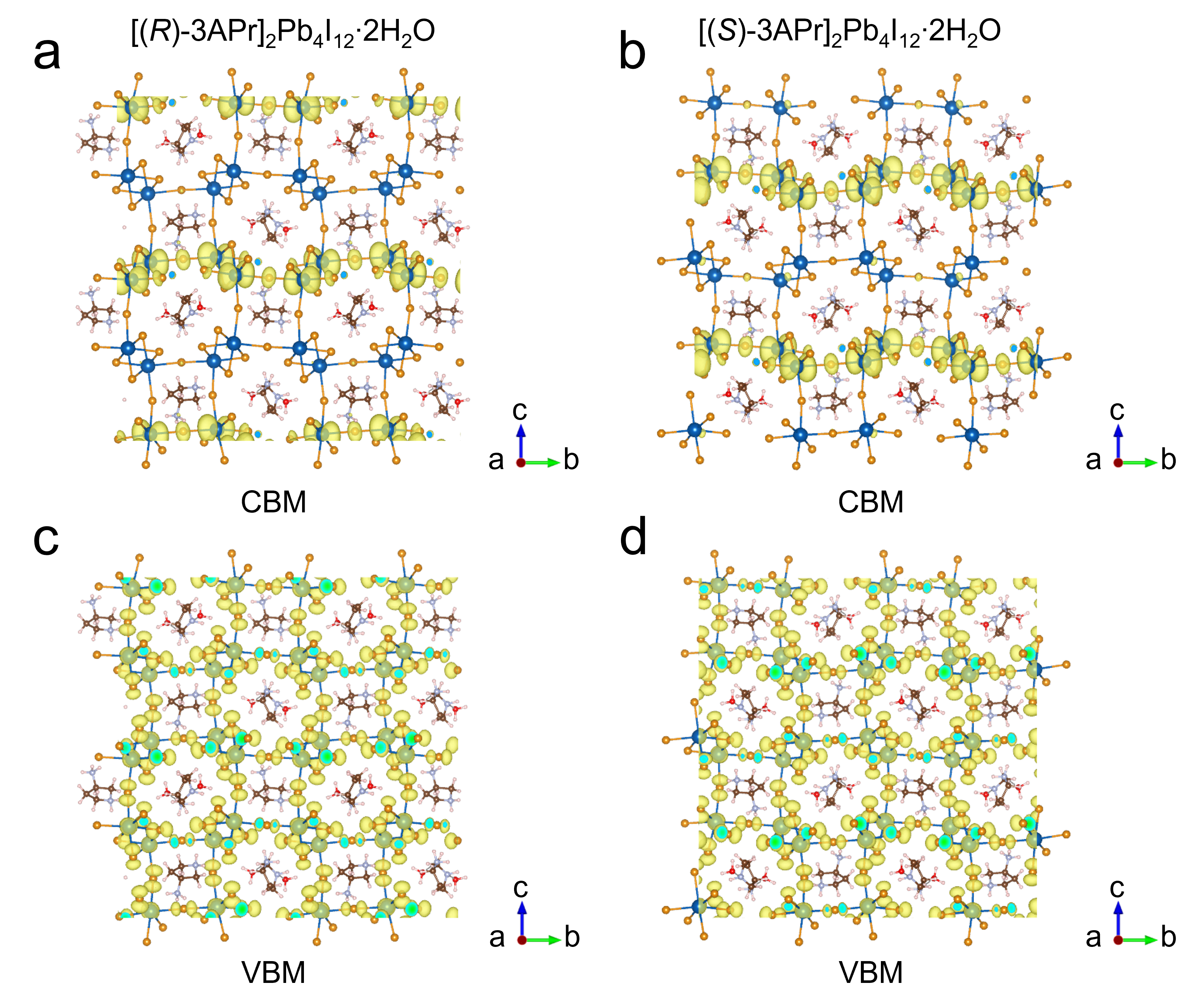


**Figure S8.** Isosurface plots of the wave functions of valence band maximum (VBM) and the conduction band minimum (CBM) of (a) [(*R*)-3APr]_2_Pb_4_I_12_·2H_2_O perovskite and (b) [(*S*)-3APr]_2_Pb_4_I_12_·2H_2_O perovskite.


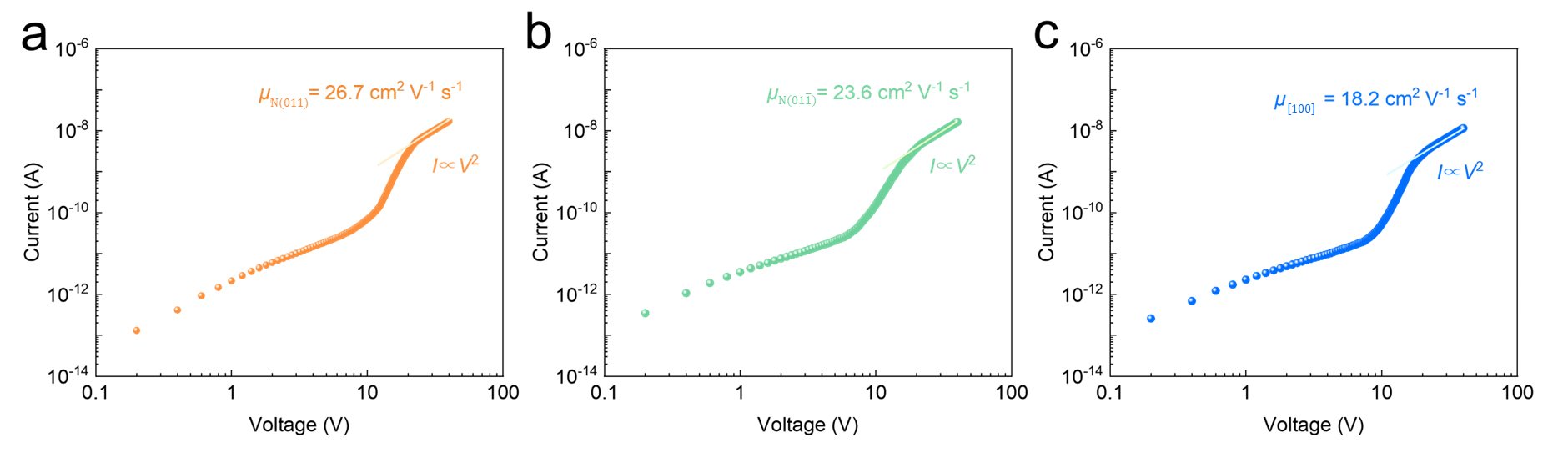


**Figure S9.** The dark current-voltage (*I*‑*V*) curves used for the carrier mobility measurement.


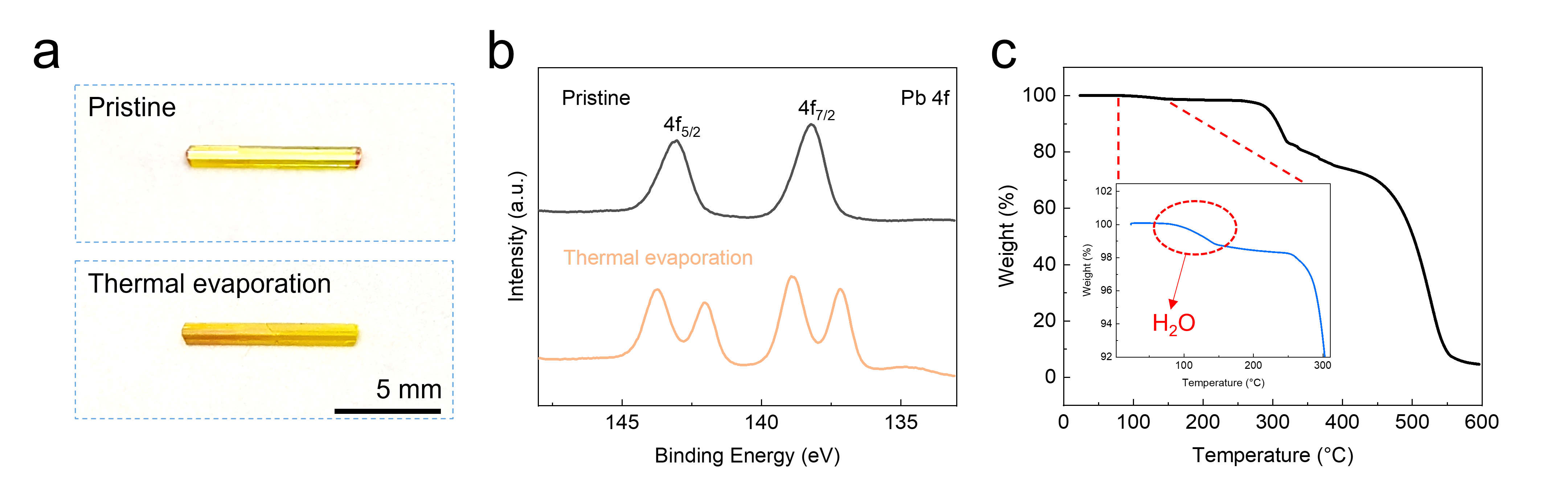


**Figure S10.** (a) Photographs of pristine single crystal and thermal evaporation-treated [(*R*)-3APr]_2_Pb_4_I_12_·2H_2_O perovskite single crystals. (b) XPS of pristine and thermal evaporation-treated [(*R*)-3APr]_2_Pb_4_I_12_·2H_2_O perovskite single crystals. (c) TGA of [(*R*)-3APr]_2_Pb_4_I_12_·2H_2_O perovskite.


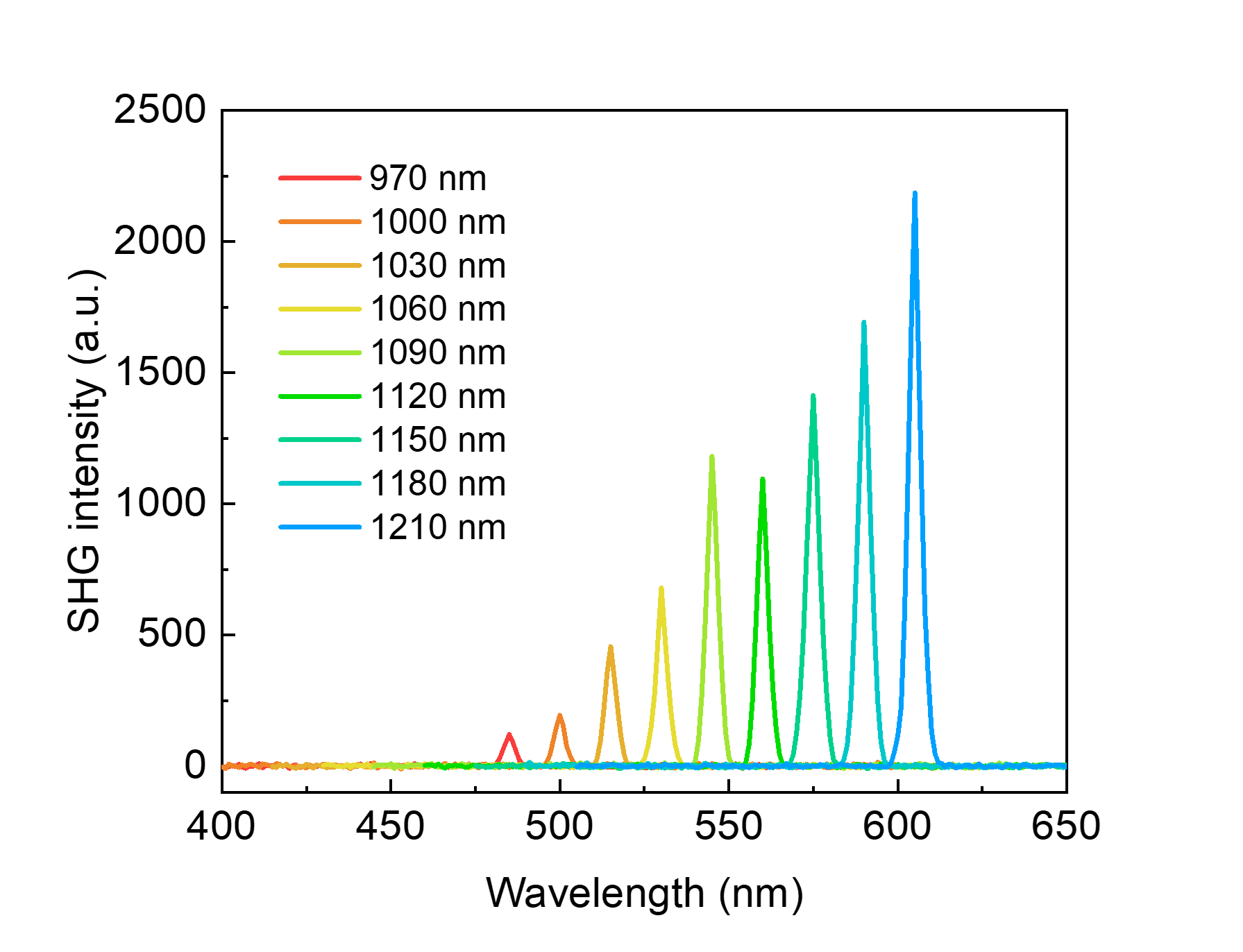


**Figure S11.** Wavelength-dependence of SHG intensities for [(*R*)-3APr]_2_Pb_4_I_12_·2H_2_O single crystal at room temperature with pump fluence of 10 mW.
